# The efficacy of toltrazuril for prevention of oriental theileriosis ( *Theileria orientalis* Ikeda genotype) in Friesian calves

**DOI:** 10.1111/avj.13415

**Published:** 2025-01-10

**Authors:** SG de Burgh, DL Emery

**Affiliations:** ^1^ The University of Sydney Sydney New South Wales 2006 Australia

**Keywords:** Ikeda genotype, oriental theileriosis, *Theileria orientalis*, toltrazuril, triazine

## Abstract

The aim of this non‐blinded, randomised, controlled study was to determine the efficacy of toltrazuril for the prevention of oriental theileriosis in calves. Thirty calves were infected with *Theileria orientalis* Ikeda genotype through the application and retention of infected female bush ticks (*Haemaphysalis longicornis*). 15 Group 1 calves were treated with a single, oral dose of toltrazuril at the dose registered in Australia for coccidiosis (15 mg/kg), concurrently with tick infection, and 15 Group 2 (control group) calves were untreated. There were no significant differences (*P* > 0.05) in mean parasitaemia, packed cell volume (PCV) or bodyweight between the toltrazuril treated and untreated groups at any timepoint up to Day 64 after infection, apart from a higher mean PCV for the toltrazuril treated group at Day 20. In this study, the calves treated with a single oral dose of toltrazuril (15 mg/kg) at the time of infection were not prevented from becoming infected with oriental theileriosis.

Pathogenic genotypes of the apicomplexan protozoal parasite, *Theileria orientalis*, were first detected in Australian cattle in 2006,^1^ and since that time, oriental theileriosis has become endemic in the geographical distribution of its principal vector, the bush tick, otherwise known globally as the Asian longhorned tick, *Haemaphysalis longicornis* Neumann, 1901.^2^, ^3^, ^4^ Ikeda has been identified as the most pathogenic genotype of *T. orientalis* in Australia.^5^

Ever since oriental theileriosis has become endemic in Australia, the two main groups of cattle significantly affected by the disease are young calves in endemic areas, and naïve cattle introduced from nonendemic to endemic areas.^6^, ^7^ Clinical signs of the disease include anaemia, diarrhoea, lethargy and mortality in calves, or jaundice, loss of production and abortions in adult cattle introduced from nonendemic areas, or under stress such as in transportation or pregnancy.^4^ The indirect lifecycle of *T. orientalis* (Figure 1) involves cattle being the intermediate host and the tick as the vector and final host.

**Figure 1:**
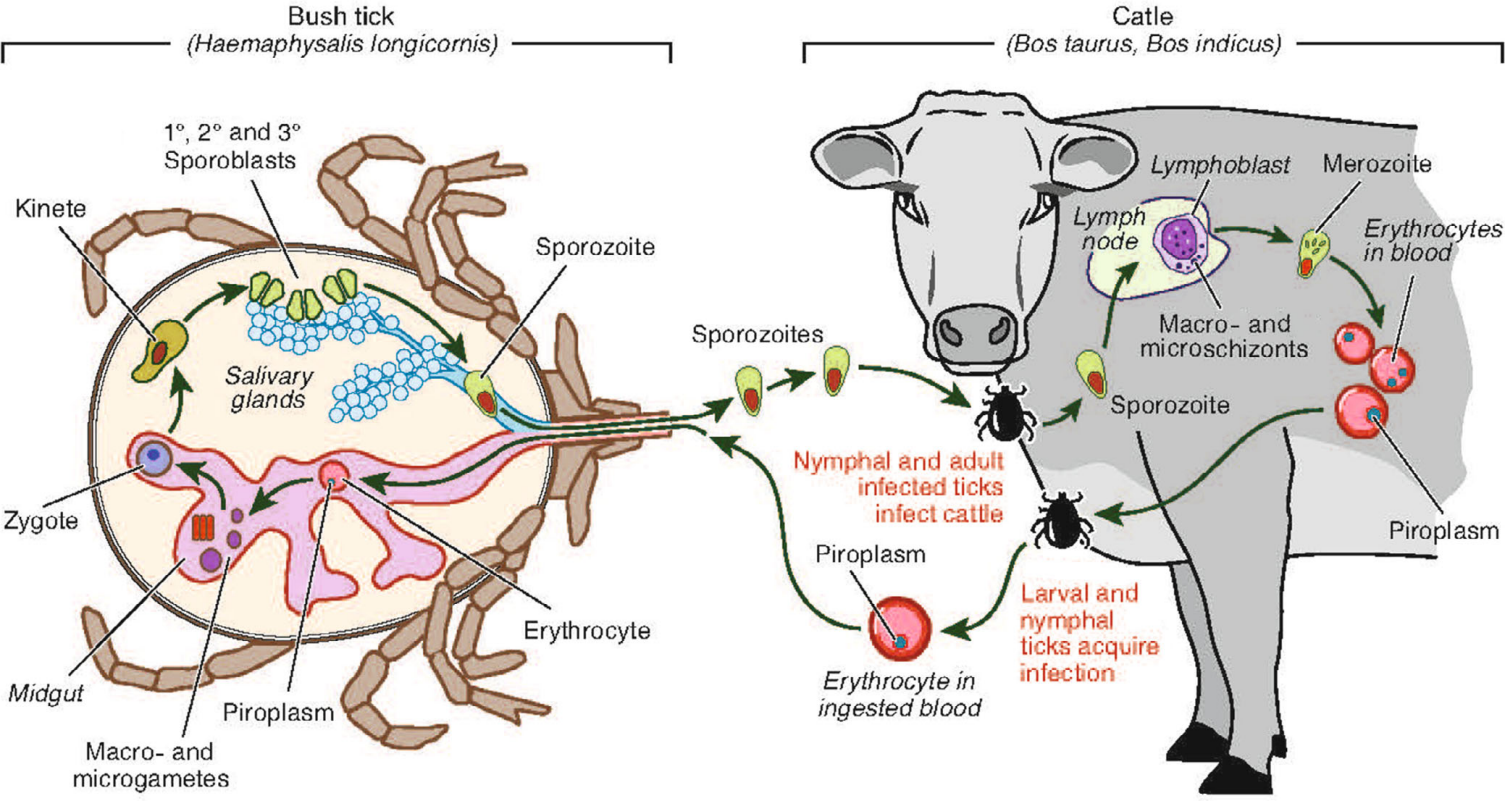
The indirect life cycle of *Theileria orientalis*. Drawings and artistic creation by Marcus Cremonese.

In 2015, financial loss to the Australian beef and dairy industries due to oriental theileriosis was estimated at $20 m pa.^8^ The disease is also recognised as an economically important parasite of cattle in New Zealand, Asia, Europe and recently the USA.^9^, ^10^, ^11^, ^12^, ^13^, ^14^, ^15^ The original goal in the US was to eradicate the introduced Asian Longhorned tick. However, as the tick has now been identified in over 15 states, eradication is no longer considered feasible.^16^ Despite significant research efforts globally to provide cattle producers with treatment and control approaches, many countries, including Australia, do not have a registered chemotherapeutic agent for the treatment of oriental theileriosis.

Buparvaquone (BPQ), a napthoquinone, that was originally developed for treatment of *Theileria parva*, was found to decrease parasitaemia and clinical signs of oriental theileriosis in infected cattle^17^ and allowed cattle to remain immune carriers of the disease after recovery.^18^ However, tissue residue studies found detectable BPQ in meat, back fat and perirenal fat up to 119 days post‐treatment and in liver, kidney and neck muscle (site of administration) up to 147 days post‐treatment.^19^ Milk residue studies found residues in milk at least 35 days after treatment.^20^ Without further studies to set minimum residue levels (MRLs) for BPQ, the long duration of detectable residues in milk and meat has made its use economically non‐viable in many countries.

Repurposing medicines registered for one indication for use against similar pathogens, or in additional species is a common method of drug development. Existing medicines have been shown to be safe, through extensive toxicological testing. The cost of drug development is greatly reduced when existing medicines can be shown to be effective for the treatment of other diseases, or in other species.^21^

In contrast to bacterial pathogens, apicomplexan parasites such as *T. orientalis* are eukaryotic and share many metabolic pathways with their eukaryotic hosts, including mammals and birds. This makes therapeutic target development difficult as drugs impacting apicomplexan parasites may also harm the host. Biomedical research to identify control methods for apicomplexan parasites is also challenging due to the complexity of maintaining live parasite cultures in the laboratory and the genetic manipulation of these organisms.^22^ There are no in vitro culture systems available for any developmental stage of *T. orientalis*.^22^ However, the recent whole genome sequencing of several *T. orientalis* genotypes and development of a mouse model of infection are building blocks towards the goal of identification of chemotherapeutic targets and vaccine candidates.^22^, ^23^

Coccidia are parasitic eukaryotes that share the phylum Apicomplexa with Theileria. Toltrazuril has been shown to have highly specific actions against several protozoa, including coccidia, in the apicomplexan group of organisms.^24^ Toltrazuril (TOL), a symmetrical triazinetrione, originally developed to treat coccidiosis in poultry, has been marketed since the end of the 1980s. In the 1990s, it was developed for use in mammalian indications, including swine and cattle.^25^ In the case of coccidia, studies have shown that toltrazuril is highly effective metaphylactically and in addition does not impact the development of host immunity, which is essential in the control of coccidiosis in intensively farmed livestock.^26^, ^27^, ^28^

Although the mechanism of action of triazines on protozoa is unclear, they have been found to inhibit metabolic enzymes and decrease pyrimidine synthesis within the specialised organelle unique to the apicomplexans, the apicoplast.^29^ Following oral administration, TOL is absorbed and rapidly converted to its metabolite, toltrazuril sulfoxide, and then metabolised to toltrazuril sulfone. These metabolites have a long terminal half‐lives, consequently enabling a long‐term clinical efficacy in the treatment of coccidiosis in calves.^30^, ^31^ In addition, it has been shown that metaphylactic treatment of calves with TOL results in simultaneous development of protective immunity to coccidia.^32^, ^33^

Baycox™ Coccidiocide for Piglets and Cattle (Toltrazuril 50 mg/mL, Elanco Australasia Pty Ltd, Macquarie Park) is registered in Australia for the treatment and prevention of coccidiosis in cattle at a dosage of 15 mg/kg, administered once orally. Just as long acting oxytetracycline has been used to attenuate *T. parva* infection by compromising the schizont stage of the parasite, anecdotal evidence from the Dorrigo area of NSW has indicated that TOL may have a prophylactic, metaphylactic and therapeutic effect on oriental theileriosis.^34^ TOL treatment may also allow in the simultaneous development of protective immunity for oriental theileriosis, as occurs with TOL treatment of coccidiosis in calves.

The aim of this study was to determine if toltrazuril, when used at the dose registered to control coccidiosis (15 mg/kg), prevented *T. orientalis* Ikeda genotype infection in calves.

## Materials and methods

In a non‐blinded, randomised study, 30 Friesian calves of mixed sex, aged between 13 and 19 weeks of age, were sourced from a commercial dairy farm. On Day −8, calves were weighed and examined by a veterinarian. Throughout the trial, calves had free access to potable water and quality pasture hay. The ration was supplemented with urea‐free calf pellets (minimum 11.25 MJ/kg DM, 16% protein, 1.2% calcium and 0.6% phosphorus) at 0.5%–1% lean body weight per day. Animals were handled in compliance with the University of Sydney Animal Ethics Committee approvals 2018/1434, 2019/1517.

**Figure 2:**
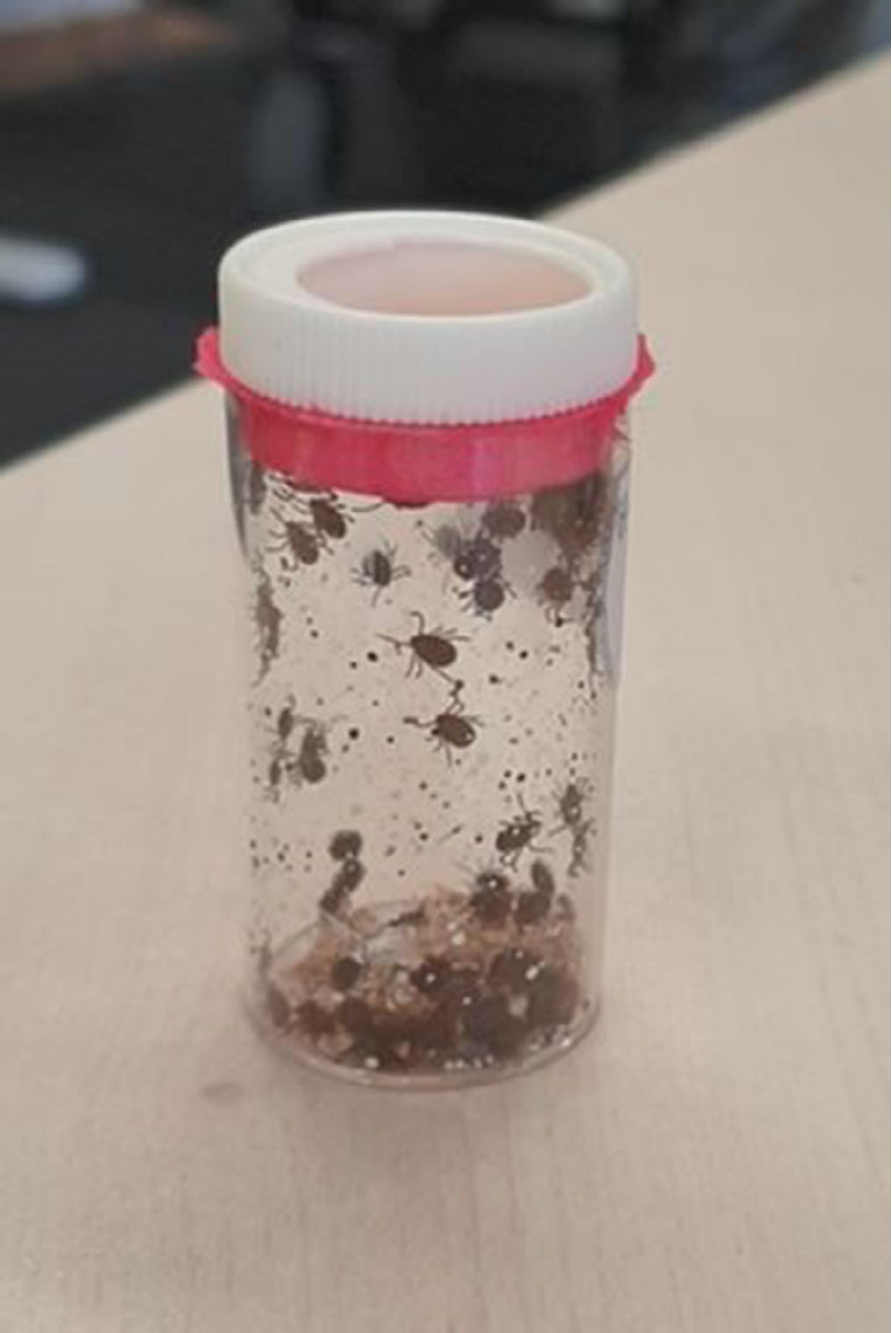
100 unfed *Theileria orientalis* Ikeda genotype infected female bush ticks for application.

**Figure 3:**
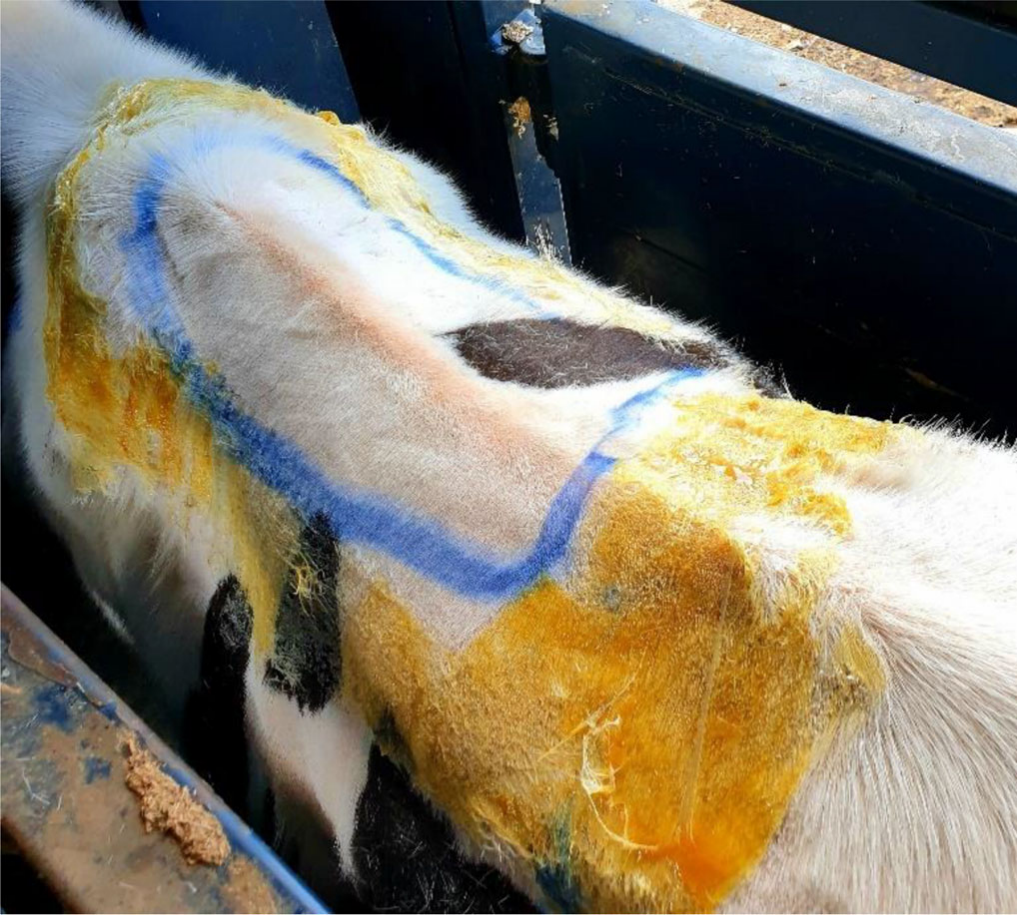
Patch template and glue application to dorsum of calf.

**Figure 4:**
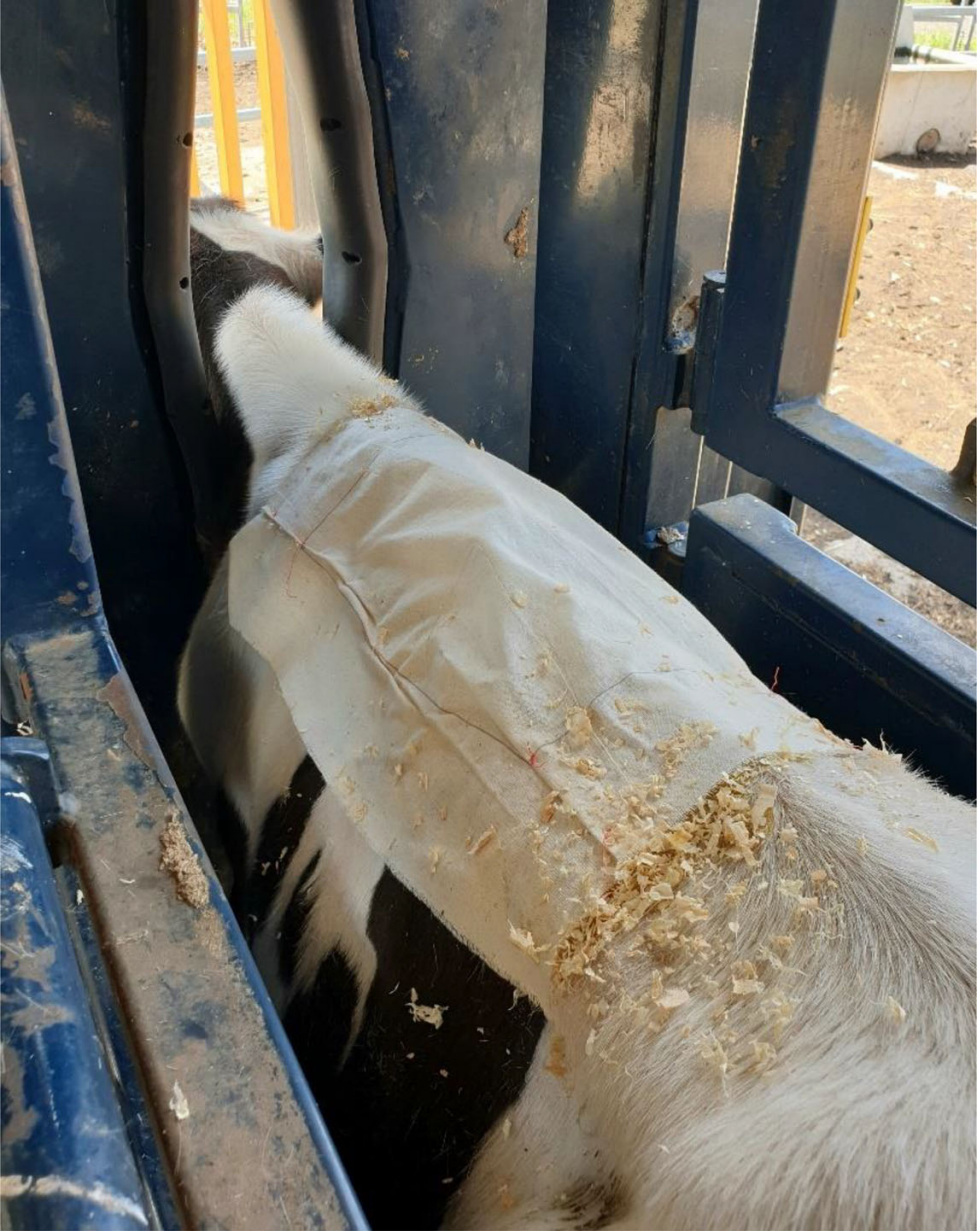
Patch application with wood shavings to contain excess glue.

**Figure 5:**
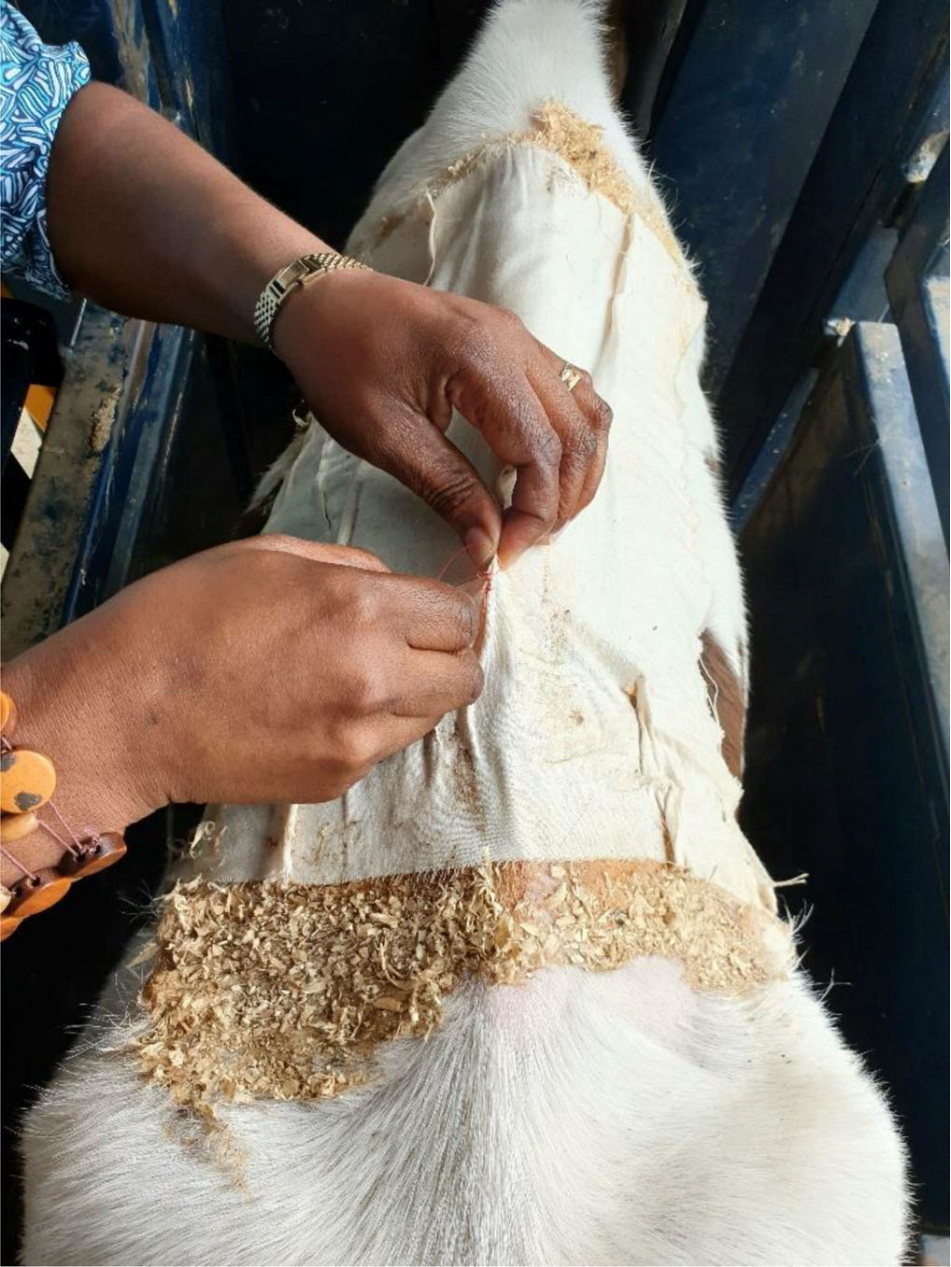
Sewing up patch after tick application.

Blood was collected on Day −1 by jugular venipuncture. Three calves were found at this time to be pre‐infected with *T. orientalis* Ikeda genotype, by quantitative polymerase chain reaction (qPCR), and were removed from the trial. Also on Day −1, the hair was shaved, and organic cotton calico patches (42 × 33 cm) were glued on to the skin, caudal to the shoulder blades of the remaining calves. The patches were sewn gathered in the corners so they would provide a raised pouch area in the centre to retain ticks during infestation (Figures 2, 3, 4, 5). Following hair clipping, a template was used to mark the area for application of Kamar® Heatmount Detector Adhesive (Agri‐gene, Wangaratta, VIC), with a 40 mm contact area around the edge of the patch. This allowed the tick feeding area under the patch to be approximately 30 × 25 cm. Clean wood shavings were applied to the edges of the patches post ‐application to contain any excess glue. Calves were closely monitored for 1 h after patch application for any signs of discomfort or removal attempts. The glue was allowed to dry for 24 h.

On Day 0, 100 adult unfed female bush ticks (*Haemaphysalis longicornis*), infected with *T. orientalis* Ikeda genotype, were applied to each calf through a hole cut in the centre of the tick patch. Infected ticks were obtained from the Tick Fever Centre, Wacol, Qld, where they had been infected through feeding on a *T. orientalis* Ikeda genotype‐positive splenectomised calf. Following tick application, patch holes were sewn up and covered with tape to ensure tick retention.

Calves were ranked by bodyweight and allocated to two groups by coin toss on Day 3. As the calves were predominantly male, randomisation was not conducted for sex. 15 (Group 1) calves were treated orally with TOL (Baycox™ 50 mg/mL) at the registered dose for the control of coccidiosis (15 mg/kg), and 15 (Group 2) calves were untreated.

Patches were opened on Day 6 and infected ticks (engorged, live or dead) were removed manually using tweezers, counted and placed in 70% ethanol. Calves were observed daily from their day of treatment until 64 days post‐treatment for clinical signs of adverse events. At each blood sampling point, animals were examined for clinical signs of oriental theileriosis.

Venous (jugular) blood samples from each study animal were obtained on Days −1, 6, 13, 20, 23, 28, 38, 41, 48, 55, and 64. Blood samples were collected into 10 mL EDTA blood tubes using 18‐gauge, 1‐inch needles. Samples were drawn up into microhaematocrit tubes to determine packed cell volume (PCV), which was used to identify the presence of anaemia (PCV ≤24). Blood was analysed for the presence of *T. orientalis* Ikeda genotype by qPCR, from samples taken on Days −1, 6, 13, 20, 23, 28, 38 and 41, as described by Bogema et al.,^35^ and Emery.^36^ Relative gene copy numbers per microlitre of blood (GC/μL) were determined for the *T. orientalis* Ikeda genotype in each sample, based on comparison with spiked standards run for each assay. Infection level is considered low and subclinical when GC/μL <15,000. Moderate infection is reported when GC/μL is between 15,000 and 300,000, and high, and likely to correspond to clinical signs of oriental theileriosis, when GC/μL >300,000.^35^

Bodyweight was measured on Days −8, −1, 3, 28, 38, 55, and 64. Group means were compared between treatment and control groups at each weighing. The schedule of trial events can be found in Table 1.

**Table 1:** Schedule of events

Study day	Clinical examination (± body weight)	Blood collection	Tick patch application	Tick application	Toltrazuril treatment	Tick removal
−8	☑					
−1	☑	☑	☑			
0	☑			☑		
3	☑				☑	
6	☑	☑				☑
13, 20, 23, 28, 38, 41, 48, 55, 64	☑	☑				

### Statistical analysis

Group means, standard errors and identification of significant differences between treated and untreated groups were identified using the students t‐test for the following key parameters: qPCR (gene copies of *T. orientalis* Ikeda genotype/μL blood), PCV (%) and bodyweight (kg), calculated in Microsoft EXCEL 365. The number of animals required per group to provide sufficient statistical power was determined by experience conducting similar studies, and significance level was set at 5%.

## Results

### *qPCR gene copies of* T. orientalis *Ikeda genotype/μL blood (GC/μL)*

All calves were found to be infected with *T. orientalis* Ikeda genotype by Day 13 post infection by qPCR. Level of parasitism was initially considered low (<15,000 GC/μL) in most calves; however, by Day 23, all calves level of parasitism was considered moderate (15,000–300,000 GC/μL) or high (>300,000 GC/μL). By Day 41 postinfection, the *T. orientalis* Ikeda genotype GC/μL had dropped to the low level for two of the infected calves; however, the remainder retained moderate to high parasitaemia.

**Figure 6:**
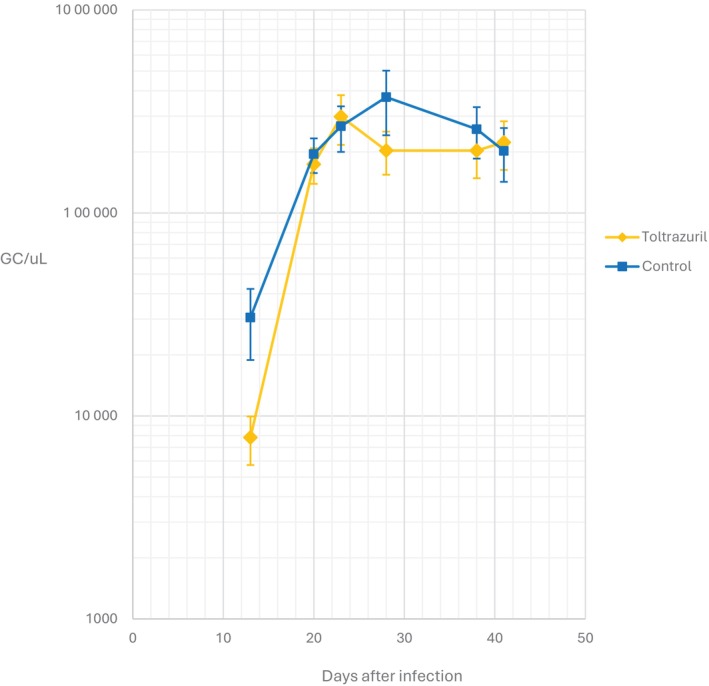
Log mean gene copies per μL of blood (GC/μL) of toltrazuril treated and control calves following infection with *Theileria orientalis* Ikeda genotype.

There were no significant differences (P < 0.05) between the treated and untreated group mean GC/μL *T. orientalis* Ikeda genotype at any sampling date post infection (Figure 6).

### Packed cell volume (PCV)

**Figure 7:**
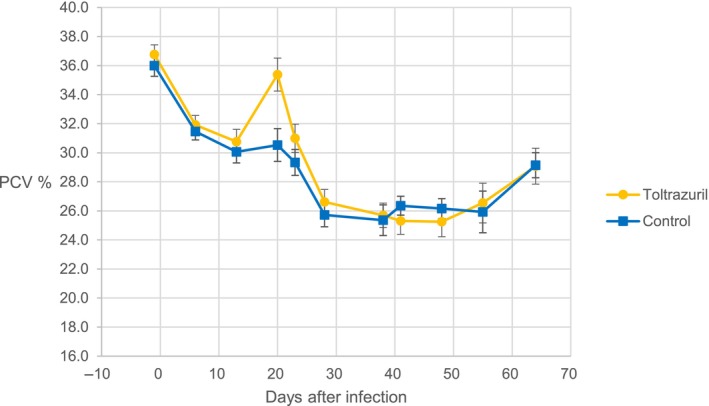
Mean packed cell volume (PCV %) of toltrazuril treated and control group calves following infection with *Theileria orientalis* Ikeda genotype.

The mean PCV for infected calves in treated and untreated groups dropped from 36.8 to 25.3 over the course of the trial. There were no significant differences between group mean PCV (P > 0.05) at any sampling date, except at Day 20. On Day 20, the mean PCV for the toltrazuril treated group was significantly higher than the untreated group (Figure 7).

Despite the group means not falling below the clinical level indicating anaemia (PCV ≤24), only 6 of the trial calves did not become anaemic at any point over the course of the trial.

### Body weight (kg)

**Figure 8:**
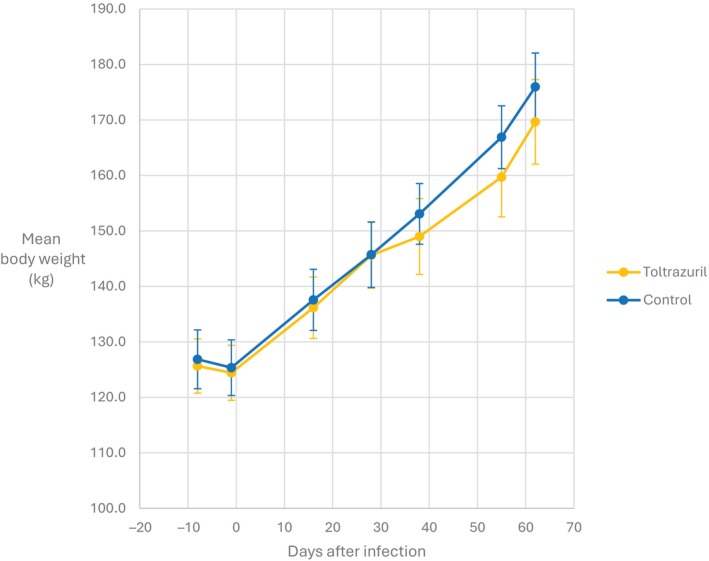
Mean body weight of toltrazuril treated and control group calves, following infection with *Theileria orientalis* Ikeda genotype.

There were no significant differences in mean body weights of treated and untreated groups at any timepoint (Figure 8).

## Discussion

In this non‐blinded, randomised trial, the infection method for *T. orientalis* Ikeda genotype in calves proved successful. All calves exposed to *T. orientalis* Ikeda genotype infected bush ticks became infected, as indicated by qPCR. The level of parasitaemia became moderate to high (>15,000 GC/μL) for all calves over the course of the trial period. Day 3 post‐tick application was chosen to administer toltrazuril, as sporozoite inoculation from ticks has been shown to be increasing from this point,^7^ and the half‐life of 8 days for toltrazuril was hypothesised to impact the production of the schizont stage of the parasite.

Prior to infection, 8 calves presented with mild cough and nasal discharge, which resolved without treatment. During the trial, possibly due to thick bushfire smoke, close contact between calves and immune compromise due to *T. orientalis* infection, 19 calves developed unilateral or bilateral infectious bovine keratoconjunctivitis (*Moraxella bovis*). Affected calves were treated with cloxacillin (Elaclox DCX Dry Cow Intramammary Suspension, Norbrook Laboratories, Tullamarine, 3043, 600 mg/5.4 g, 2 mL into the subconjunctiva) as required. Five calves developed corneal ulceration as sequalae to *M. bovis* infection and were treated with debridement under local anaesthetic (Ilium Lignocaine 20 Local Anaesthetic Injection, Troy Laboratories, Glendenning 2761, lignocaine hydrochloride 20 mg/mL, 1 mL by subcutaneous injection into the eyelid) and parenteral antibiotics (Ilium Propercillin Antibiotic Injection, Troy Laboratories, Glendenning 2761, procaine penicillin, 300 mg/mL, 2 mL by subcutaneous injection into the eyelid). Six calves developed superficial facial ringworm lesions (*Trichophyton verrucosum*) during the trial, which were not treated.

In addition, during the trial period, two of the infected calves were euthanased due to poor health. One was identified as pre‐infected with the *T. orientalis* Ikeda genotype prior to tick infection and was removed from the trial. The second was one of the youngest and lightest of the calves enrolled in the trial. This calf presented initially with diarrhoea prior to infection and weight loss post infection. Data from these calves were removed from analysis.

In order to abate the clinical signs of oriental theileriosis, all calves received supportive treatment with parental multivitamin and mineral injections on Days 24 and 54 (VitADE Injection, Abbey Laboratories Glendenning 2761, 4 mL by intramuscular injection, and Multimin Injection for Cattle, Virbac Australia, Milperra, 2214, 1.5 mL by subcutaneous injection). Also, as supportive treatment, on Day 24 all calves were treated with trimethoprim sulfoxidine (Trivetrin Injection, Intervet Australia, trimethoprim 40 mg/mL, sulfoxidine 200 mg/mL, 6 mL by intramuscular injection) and on Day 54 with oxytetracycline (Alamycin LA 300 Oxytetracycline Injectable Solution, Norbrook Laboratories, Tullamarine, 3043, oxytetracycline 300 mg/mL, 15 mL by intramuscular injection).

Immune compromise due to age of calves, environmental conditions and infection with oriental theileriosis impacted the health of calves enrolled in this trial. As all calves in the toltrazuril treated and untreated groups received the same animal husbandry and supportive treatments, and co‐morbidities encountered by calves were approximately evenly distributed between groups, they were determined not to impact the results of the trial.

Mean parasitaemia measured by qPCR (GC/μL) was not significantly different between the toltrazuril treated and untreated groups at any timepoint during the trial period (Figure 6). The variance between animals within groups peaked in the control group at Day 28 after infection.

Clinical anaemia (PCV ≤24) was detected over the trial period in all but 6 calves, indicating that the infection level instigated by the application of 100 infected female bush ticks was clinically significant. There were no statistically significant differences in group mean PCV at any timepoints, except at Day 20, where the mean PCV of the toltrazuril‐treated group was significantly higher that the untreated group (P < 0.05). Even though toltrazuril has a half‐life of only 8 days in cattle,^30^ treatment may have initially suppressed the clinical signs of infection. However, PCV at subsequent timepoints, and qPCR at all timepoints were not significantly different between groups, indicating no prolonged impact of toltrazuril on oriental theileriosis at the registered dose used for the control of coccidiosis.

In a study conducted in New Zealand, calves from herds naturally infected with the *T. orientalis* Ikeda genotype were treated with a single dose of toltrazuril at the registered dose rate for coccidiosis (15 mg/kg), at 4 weeks post turn‐out onto pasture.^37^ In this study, levels of parasitaemia remained subclinical in all calves, and no significant differences were found between treated and untreated groups when monitored over 16 weeks post turn‐out. This study indicated that treatment with toltrazuril (15 mg/kg) up to 3 weeks after infection had no significant impact on the level of oriental theileriosis parasitaemia.

The goal regarding management of oriental theileriosis in endemic areas is for infected cattle to reach the carrier state without parasitaemia rising to the high levels that lead to clinical signs of disease. The carrier state is protective against new infection from subsequent infected tick bites. Ideal chemotherapeutics for oriental theileriosis will limit infection to subclinical levels and allow the development of the carrier state without impacting production.^38^, ^39^, ^40^, ^41^ Treatments such as BPQ may reduce the first peak of parasitaemia so animals can reach the carrier state, as found in a recent study.^18^

In conclusion, this study showed that toltrazuril, when used at the dose registered to control coccidiosis (15 mg/kg), was not effective prophylactically, metaphylactically or therapeutically for *T. orientalis* Ikeda genotype prevention in the calves enrolled in this trial. There was, however, one timepoint (Day 20) where the mean PCV for the toltrazuril group was higher than the untreated group, but this effect was not identified in the level of parasitaemia, measured as GC/μL by qPCR.

## Conflicts of interest and sources of funding

Susan de Burgh was an employee of Bayer Australia Pty Ltd at the time of this research, and the funding for the research was shared by Bayer and the University of Sydney.

## Data Availability

The data that support the findings of this study are available on request from the corresponding author. The data are not publicly available due to privacy or ethical restrictions.
